# Comparative Population Dynamics of Two Closely Related Species Differing in Ploidy Level

**DOI:** 10.1371/journal.pone.0075563

**Published:** 2013-10-07

**Authors:** Lucie Černá, Zuzana Münzbergová

**Affiliations:** 1 Department of Botany, Faculty of Science, Charles University, Prague, Czech Republic; 2 Institute of Botany, Academy of Sciences, Průhonice, Czech Republic; Beijing Forestry University, China

## Abstract

**Background:**

Many studies compare the population dynamics of single species within multiple habitat types, while much less is known about the differences in population dynamics in closely related species in the same habitat. Additionally, comparisons of the effect of habitat types and species are largely missing.

**Methodology and Principal Findings:**

We estimated the importance of the habitat type and species for population dynamics of plants. Specifically, we compared the dynamics of two closely related species, the allotetraploid species *Anthericum liliago* and the diploid species *Anthericum ramosum*, occurring in the same habitat type. We also compared the dynamics of *A. ramosum* in two contrasting habitats. We examined three populations per species and habitat type. The results showed that single life history traits as well as the mean population dynamics of *A. liliago* and *A. ramosum* from the same habitat type were more similar than the population dynamics of *A. ramosum* from the two contrasting habitats.

**Conclusions:**

Our findings suggest that when transferring knowledge regarding population dynamics between populations, we need to take habitat conditions into account, as these conditions appear to be more important than the species involved (ploidy level). However, the two species differ significantly in their overall population growth rates, indicating that the ploidy level has an effect on species performance. In contrast to what has been suggested by previous studies, we observed a higher population growth rate in the diploid species. This is in agreement with the wider range of habitats occupied by the diploid species.

## Introduction

Understanding the population dynamics of a species is a key prerequisite when attempting to understand the factors determining the performance of its populations [1], [2], [3]. Most studies addressing the population biology of various plant species investigate specific life-cycle stages or single traits, such as seed production, seedling survival or population size (e.g., [4], [5], [6], [7], [8], [9], [10], [11]). However, to understand the causes of changes in population size, it is necessary to investigate the complete life cycle of a species, as it is only by putting the various life-cycle transitions into the context of the whole life cycle that we can understand the consequences of the differences in single life history traits for the dynamics of a population (e.g., [12], [13], [14], [15], [16], [17], [18], [19]).

Performing a detailed analysis of the entire life cycle of a species is a demanding task, and it is not possible to perform such analyses for all species of interest. It would therefore be useful to be able to transfer knowledge obtained from one population and/or species to other populations of the same species and/or different species. To evaluate this possibility, we need to understand the differences in the population dynamics of closely related species in the same habitat type and of individual species in different habitat types. However, such knowledge is still largely lacking (but see [15], [20], [21], [22]).

One of the types of species that is suitable for studying the dynamics of closely related species and obtaining information about the minimum differences in the population dynamics of closely related species are pairs of diploid and polyploid species. Polyploidy is relatively frequent in the plant kingdom, and it is estimated that between 47 and 70% of all plant species are polyploid [23]. More recent estimates even suggest that all angiosperms have undergone at least one polyploidization event [24]. Understanding the consequences of polyploidization for the population dynamics of plants is therefore of major importance and can help us to evaluate the consequences of polyploidization events for species diversity. Studies comparing the behavior of diploid and polyploid species have usually concentrated on individual stages of the life cycle, e.g. germination and seedling recruitment (e.g. [25], [26]) and individual growth rates, inflorescence production and flower morphology (e.g. [27], [28], [29], [30]). Only few studies have compared the complete life cycles of diploid and polyploid species. Münzbergová [15] compared the complete life cycles of diploid and autohexaploid perennial herbs and detected no overall differences in population dynamics, despite large differences in single life history traits. However, the hexaploid populations exhibited a higher extinction probability. Bucharová et al. [21] compared diploid and allotetraploid ferns and demonstrated the existence of a higher extinction probability and smaller number of populations in the diploid species. Buggs & Pannell [31] compared performance of diploid and tetraploid individuals of annual *Mercurialis annua* and demonstrated that diploid plants have higher fitness. This is in agreement with the fact that diploids are currently displacing polyploids in the area.

Another type of comparison that could help us to understand the variability of population dynamics and therefore tell us something about the possibility of transferring the conclusions of studies on the population dynamics of single species is analysis of the dynamics of a single species in different habitat types. As for different ploidy levels, many studies have compared single life history traits between populations from different habitats (e.g., [32], [33]). Recently, various authors started to compare the whole life cycle of populations of the same species occurring in different habitat types (e.g., [34], [35], [36], [37], [38], [15], [39], [40], [41], [42], [43]).

In the present study, we estimated the importance of habitat types and species (ploidy levels) in the population dynamics of plants. Specifically, we compared the dynamics of two closely related species, *Anthericum liliago* (4n) and *Anthericum ramosum* (2n), in the same habitat type in the same localities as well as the dynamics of *A. ramosum* in two contrasting habitats (forest and open habitat). Specifically, we asked the following questions. (1) What are the differences in single life history traits between the two species and between populations of a single species in different types of habitat? (2) How do these differences translate into differences in population growth rates? (3) Are the observed differences between species comparable to the differences between habitats within a single species?

Studies in polyploid species often suggest that species with higher ploidy levels perform better than their diploid ancestors because they possess a greater number of different alleles and therefore are able to adapt to different environments better (e.g., [44], [45], [46], [47], [48], [49], but see [50]). Based on this notion, we predicted that the tetraploid species *Anthericum liliago* perform better than the diploid species *Anthericum ramosum*.

## Materials and Methods

No specific permissions were required for entering the localities and for dealing with the species as all the performed studies were only observational and did not involve any plant destruction.

### Study Species

*Anthericum liliago* L. (AL) and *Anthericum ramosum* L. (AR) (Asphodelaceae) are closely related, long-lived perennial herbs that are typical of dry grasslands and that exhibit frequent clonal reproduction. *A. liliago* is an allotetraploid (2n = 60), and *A. ramosum* is one of its diploid (2n = 30) progenitors [51]. These species are able to produce triploid hybrids. Triploid populations are known from Scandinavia [51]. However, the existence of triploids in nature in the study region, the Czech Republic, has not been confirmed ([52], our unpubl. data). The absence of hybrids in the Czech Republic may be at least partly attributed to the fact that the two species are phenologically separated. *A. liliago* flowers from May to early June, while *A. ramosum* begins flowering in June and continues until August [53]. The distribution range of both species in Europe extends from Spain across France and central Europe to the Balkans and Ukraine. In the north, their distribution range reaches southern Sweden and the Baltic states. In the Czech Republic, *A. ramosum* can be found in both Bohemia (western part) and Moravia (eastern part). For *A. liliago*, the eastern boundary of the distribution range is in central Bohemia.

In the Czech Republic, the two species usually occur separately. *A. liliago* prefers open, sunny stony slopes and rocks with a southern exposure. *A. ramosum* occupies sunny hillsides (often on a calcareous substrate) and dry open forests. On some open sunny slopes, these two species co-occur. In these habitats, it is possible to compare the population dynamics of the two species under the same habitat conditions and, thus, detect minimum differences in the population dynamics of two closely related species with different ploidy levels. Furthermore, the different habitat types occupied by *A. ramosum* allow differences in the dynamics of populations of a single species to be studied in different environmental conditions. Unfortunately, it is not possible to set up a full factorial study in this system, as *A. liliago* does not occur in forests.

### Study Localities

**Figure 1:**
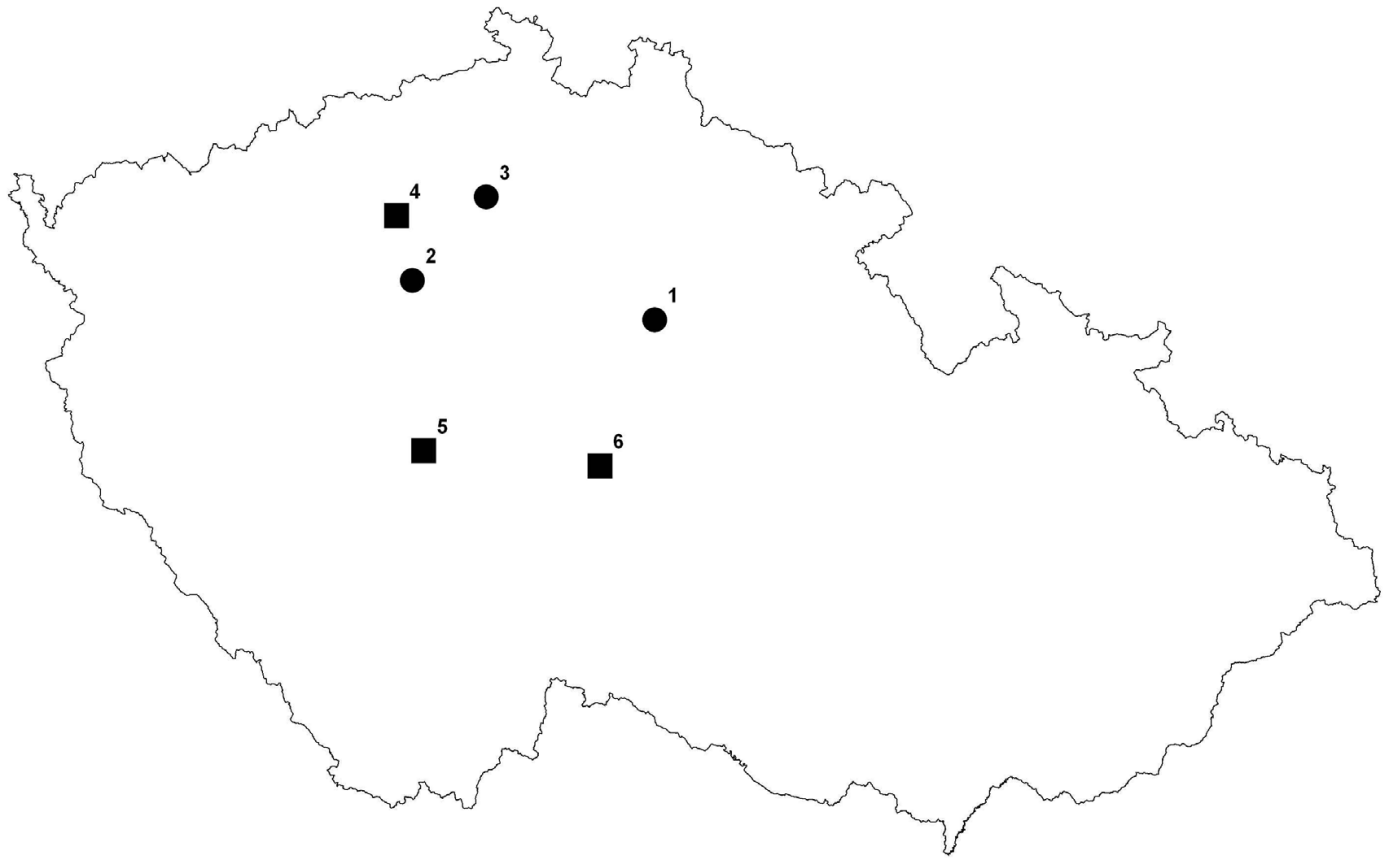
A map of the studied localities in the Czech Republic, Europe. Circles indicate the open localities (hosting *A. liliago* as well as *A. ramosum* populations) and squares indicate the forest localities (hosting only *A. ramosum* populations).

To compare the population dynamics of *A. liliago* and *A. ramosum*, we selected 3 localities in open habitats where the two species co-occur in the Czech Republic. To study the population dynamics of *A. ramosum* in contrasting habitats, 3 additional forest localities were chosen. Therefore, 6 localities and 9 populations were studied in all (Figure 1).

At each locality in each studied population, the basic habitat conditions were investigated separately for patches where *A. liliago* and *A. ramosum* occurred. The populations of the two study species never completely overlap in the open localities, so we selected patches with only one or the other species for comparison of the habitat conditions. For each locality and species, the vegetation composition was assessed in 3 replicates and the soil conditions in 5 replicates. There were no differences observed in the vegetation composition or soil conditions between patches containing the two species within a single locality, suggesting that the microsites occupied by the two species do not differ (Figures S1, S2 and S3).

### Population Dynamics

The complete life cycles of both species were described by marking at least 150 individuals (ramets) in each studied population in permanent plots (5 plots per population, with approximately 30 marked individuals per plot). Each individual was marked with a metal label placed just beside the plant. All individuals in each plot were marked to allow clonal growth to be investigated. If the number of individuals in a certain stage was too small in the overall sample from the given population (less than 30), we marked additional individuals at that stage outside the plots to ensure that we had approximately equal numbers of individuals marked in each stage [54].

To describe plant growth, we recorded the presence of flowering stalks, the numbers of leaves and the length of the longest leaf on each plant during peak flowering in June for *A. liliago* and in August for *A. ramosum* in the open habitats and at the beginning of September for *A. ramosum* in the forest habitats. The differences in the recording times were due to phenological differences between the species and habitats. Additionally, all new clonal ramets and seedlings were recorded and marked every year in April in the open habitats and in May in the forest, i.e., at the time when it was possible to reliably distinguish seedlings from new clones. Seedlings can be differentiated from young clones in the field mainly by the fact that the outer part of the seed is attached to the bottom of the seedlings in spring. Additionally, the first leaf of seedlings is convolute and can be distinguished from the first leaf of small clonal plants, which is flat. The population dynamics were studied over two transition intervals (2006–2007 and 2007–2008). Data on mean annual temperature and annul precipitation in the region in the studied years in comparison with data from a larger time period are provided in Figures S4 and S5.

To construct transition matrices, we inspected the collected data and tested the usefulness of various size parameters, mainly number of leaves, length of the longest leaf and number of leaves×length of the longest leaf considered as a proxy of total leaf biomass, for delimitating different size categories of plants. Based on this preliminary inspection, the life cycles of the plants were divided into 5 stages: seed, seedling, small vegetative, large vegetative (≥7 leaves) and flowering plants. The criteria for considering vegetative plants as small or large were based on the relationship between plant size and the probability of flowering in the next year, which indicates that plants with at least 7 leaves exhibit a much higher probability of flowering than smaller plants. New clones could be either small or large vegetative plants or could be flowering. In the field, it was not possible to distinguish to which parental plant the new clones belonged. We therefore assumed that new clones with the size of small vegetative plants could have been produced by both small and large vegetative plants as well as flowering plants; new clones with the size of large vegetative plants could have been produced by large vegetative plants and flowering plants; and new flowering clones could have been produced only by flowering plants. We then considered the new clones found in each plot as offspring of all of the appropriate maternal plants in that plot. We divided the number of new clones by the number of potential maternal plants. In this way, we obtained the number of clones of a given size category per maternal plant. Thus, we assumed that plants of different sizes produced the same number of clonal offspring, but the clonal offspring could never be larger than the maternal plant producing the offspring. These values were added to the transition probabilities obtained from following the marked individuals over time. It was possible to apply this calculation because all of the permanent plots were situated in homogenous stands of the species. It can therefore be assumed that the number of clones growing outside the plots was equal to the number of clones growing within the plots.

Generative reproduction was studied in a sowing experiment. In each population, 10 sowing plots with controls (plots without sowing to check for background natural germination) were established, and 50 seeds were sown in each plot after seed ripening in autumn 2006 and 2007. The number of germinated seedlings was counted when the demographic data were recorded in the following years. Number of seedlings in the control plots was subtracted from the number of seedlings in the sowing plots to control for background germination. For the sowing experiment established in 2006, the number of germinated seedlings was also counted 2 years later (in 2008) to identify the proportion of seeds showing delayed germination. Only a few natural seedlings (seed from natural seed rain or from seed bank) were found in permanent demography plots (probably due to a significant drought in early summer 2006). Thus, seedling survival was studied using seedlings germinated in the sowing plots in 2007. This value of seedling survival therefore had to be used for the matrices obtained from both transition intervals. Data on seedling survival were also available from 2006 for both species from the sowing plots established in 2005 at locality 1. These data were comparable with the data obtained in 2007, indicating that using data only from one year did not represent a major problem.

To examine seed survival in the seed bank, 150 seeds from each studied population were buried 5 cm underground at the studied localities for one year. The seeds were divided into three fine mesh bags, and each bag was buried approximately 1 m from the other bags to cover the variation at the locality and still be able to relocate the seeds easily. We compared the germination of these seeds with the germination of seeds before being buried underground in a growth chamber experiment. Before germination, the seeds were placed on wet filter paper in Petri dishes (9 cm in diameter, 50 seeds per Petri dish). The Petri dishes were first placed in a refrigerator (4°C) for 6 weeks for stratification, after which they were transferred to a growth chamber with a fluctuating light and temperature regime (12 hours light/12 hours dark, 20°C in light and 10°C in the dark). After three months, the non-germinated seeds were checked for viability using the tetrazolium test [55]. Because the survival of seeds in the seed bank was quite high and the seeds are hard coated, it can be expected that the species forms long-term seed bank. In the transition matrix, the proportion of seeds surviving in the seed bank represents the transition from seed to seed. We thus assume, that the same proportion of seeds survives in the seed bank each year and in this way we in fact incorporate permanent seed bank into our models.

Seed production was estimated as the mean number of seeds that developed from 20 randomly selected plants in each population in each year (the variation in seed production between individual plants was not very large, with the mean SD over the populations = 3.74).

### Data Analysis

All of the analyses were separated into two parts: comparison of populations of *A. liliago* and *A. ramosum* from the open habitats and comparison of *A. ramosum* populations from open habitats and forests. This separation was necessary because the design of the study was not fully factorial (as *A. liliago* does not form large forest populations that could be studied). To take into account the fact that the same data were always used in two independent sets of tests, the conventional α level employed to assess significance was reduced from 0.05 to 0.025 [15], [56].

First, we tested the effect of the species (using data on both species from the open habitat) and habitat (using data on *A. ramosum* from both habitats) on single species traits. In this case, the species/habitat, year, locality nested within the species/habitat and their interactions were used as independent variables. The dependent variables were the number of seedlings, probability of flowering and survival. The number of seedlings was tested using a generalized linear model (GLM), assuming a Poisson distribution. The probability of flowering and survival was also tested using a GLM, assuming binomial distribution. In this case, the model also included the stage in the previous year to take into account the fact that plants of different stages exhibit a different probability of survival and flowering. Because the individuals from a single population were not independent, the effect of the species/habitat and the interaction of the species/habitat and year or stage in the previous year were tested against the locality and locality×year or locality×stage in the previous year and not against residual variance using quasi-F statistics [57]. The effects of the locality, locality×year and locality×stage in the previous year were tested against the residual variation.

Demographic data were examined using transition matrix models [58]. Analysis of a transition matrix yields a finite rate of increase, λ, for a population. Analyses of projection matrices also generate information about the change in the population growth rate, δλ, following a small change in a given matrix element, a_ij_ (δa_ij_), referred to as the sensitivity. Proportional sensitivity, or elasticity, is usually used as a measure of the contribution of a matrix element to fitness [59]. In this study, we calculated the finite rate of increase, λ (population growth rate), as well as the elasticity for each studied population and transition interval separately. To allow the obtained patterns of elasticity to be compared with other studies, we summarized the elasticity values for survival, growth and reproduction as suggested by Silvertown et al. [60]. Survival in this case represents combination of stasis, retrogression and clonal growth as clonal growth is included in these transitions and caanot be separated (see vital rates elasticity below for separation of the importance of clonal growth).

Each estimate of the transition probability and, thus, each estimate of the population growth rate and of elasticity is confined by an error because of the limited number of individuals that can be sampled. To estimate this error, bootstrap confidence intervals [61] were calculated for the population growth rate and elasticity of each studied population. This was carried out by bootstrapping the original data used to derive the transition matrices 10 000 times. Based on the results, confidence intervals of population growth rates and elasticities were constructed for each population and year [62]. For this purpose, a MATLAB script developed for a previous study [14] was used.

Furthermore, to combine all of the matrices of one type (*A. liliago* from open habitats, *A. ramosum* from open habitats and *A. ramosum* from forest habitats) and to estimate the overall population growth rates and elasticities for these combined matrices, the stochastic simulation approach was used ([58], [63]). For each set of matrices, we drew a random sequence of matrices. Each matrix from the set was drawn at random and with an equal probability, and we simulated population growth using this matrix sequence. Each simulation was run for 10 000 one-year intervals and used for the calculation of population growth rates and elasticities. The simulations were performed using a MATLAB script developed in our previous studies ([13], [15]) We ran the same stochastic simulations for the bootstrapped matrices described above and were therefore able to construct a 95% confidence interval of the stochastic population growth rate.

In some studies (e.g. [64], [65]), the authors draw matrices for calculating stochastic population growth rate according the proportion of years with different climatic conditions. In our data, the values of precipitation vary more strongly than temperature. We thus calculated the number of years between 1961 and 2012 (i.e. the period for which climatic data are available) with precipitation above or equal to the higher value in the studied period (2007) and with precipitation lower or equal to the lower value in the studied period (2008). The resulting numbers are 20 and 14 (Figure S5). This indicates that similarly dry and similarly wet years are quite common and occur with relatively comparable proportions 20∶14. We thus decided to keep an equal proportion of years in the stochastic simulations.

A life-table response experiment (LTRE) with a fixed factorial design was conducted to examine the effect of the species/habitat, year and locality and the interaction between the species/habitat and year on the population growth rate. LTRE is a form of retrospective analysis that allows quantification of factors responsible for the observed variation in population growth rates. Compared with elasticity analysis, LTRE analysis quantifies the observed effects of single matrix elements on the observed variation in population growth rates, rather than expected effects. We followed the approach described by Caswell [58]. The LTRE analysis indicates the contribution of each life-cycle transition to the differences between the levels of each factor, i.e., the species/habitat, year, locality and the interaction between the species/habitat and year. Important life-cycle transitions are those with large positive contributions at some factor levels and large negative contributions at others. Analogous to ANOVA, the mean of the treatment effects is approximately zero [58]. The significance of the overall contribution and the contribution of each matrix element was tested using permutation tests with 1000 permutation runs. In each permutation run, each individual plant used to estimate the transition probability in the transition matrices was randomly assigned to a transition matrix. New transition matrices were then created based on these permuted data, and the LTRE analysis was repeated. Next, it was calculated how often the observed overall contribution and the contribution of each matrix element was larger than would be expected if the individuals were distributed among groups at random. The obtained value was used as a significance value to identify the matrix elements that significantly contributed to differences between the categories being compared and to estimate the significance of the overall differences between the two groups being compared. A MATLAB script was employed to perform the analysis, and 10 000 permutations were used in each case [15]. To visualize the LTRE values for the two species and habitat types, we also summed the positive and negative contributions separately for survival, growth and reproduction (similarly as we have done it for elasticity values) and plotted these values [37].

The elasticity and LTRE analyzes are standard approaches to analyze demographic data. They have, however, also been criticized because they analyze the importance of single transitions within the life cycle, rather than different vital rates ([66]). To explore the importance of vital rates in our system we partly modified the approach described in Zuidema & Franco [66]. The vital rates in our system included growth, stasis, retrogression, reproduction and clonal growth. We took the original data (in contrast to assumed distribution of the data in [66]) and bootstrapped them in the same way as when calculating confidence intervals of lambda. In each case, we, however, bootstrapped only transitions (or their parts) belonging to a given vital rate and kept all the other transition or their parts constant. Because clonal growth is part of the transitions corresponding to stasis and retrogression, we bootstrapped the different parts of the transitions separately (either only clonal growth, or stasis or retrogression, and the original value of the other part was added to the bootstrapped value for constructing the whole matrix). We used 1500 bootstrap replicates for each transition matrix and vital rate. In each case we assessed lambda of the resulting matrices and calculated CV and width of 95% confidence intervals of the resulting lambda. The CV values are termed elasticity of the vital rates and the 95% confidence intervals are termed sensitivity values of the vital rates in [66]. Because these two measures were very closely correlated in our dataset (r >0.99), we represent only the CV values, termed elasticity of the vital rates, in the results.

## Results

### Single Life History Traits

The effect of the species on seed germination was only marginally significant, with *A. liliago* showing a higher germination rate (18.33%) than *A. ramosum* in the open habitat (9.53%). The effect of the habitat type on seed germination was non-significant (*A. ramosum* in the forest exhibited 26% germination, Table 1). The non-significance of the differences between the species and habitats was due to the existence of large significant differences between years and between single localities. There were also significant interactions between the species/habitat, locality and year (Table 1). The germination of *A. liliago* did not differ greatly between years, but there was a 20% increase in the germination of *A. ramosum* in the open habitat in the second year. The differences in germination between the two years were much more apparent in the populations from the forest habitat, which showed average germination rates of 2% in the first year and 50% in the second year.

**Table 1:** Effect of the year, species/habitat, locality nested within the species/habitat, stage in the previous year and their interactions on seed germination, survival and the probability of flowering.

			Germination			Probability of survival			Probability of flowering		
		d.f.	d.f. error	R^2^	*P*	d.f. error	R^2^	*P*	d.f. error	R^2^	*P*
Species	Year	1	87	0.075	**<0.001**	2351	–	0.23	1956	–	0.71
	Stage	2	NA	NA	NA	2348	0.068	**<0.001**	1954	0.273	**<0.001**
	Species	1	4	0.007	*0.026*	4	–	0.42	4	0.020	**0.019**
	Locality	4	41	0.197	**<0.001**	1172	0.019	**<0.001**	975	0.014	**<0.001**
	Stage * year	2	NA	NA	NA	2340	–	0.60	1947	–	0.33
	Species * year	1	8	0.075	**<0.001**	8	–	0.10	8	0.013	**<0.001**
	Locality * year	4	78	0.152	**<0.001**	2332	0.026	**<0.001**	1940	0.009	**<0.001**
	Species * stage	2	NA	NA	NA	2336	0.008	**0.015**	1944	0.006	**<0.001**
	Locality * stage	8	NA	NA	NA	2320	–	0.21	1932	0.009	**0.003**
Habitat	Year	1	87	0.530	**<0.001**	2055	0.008	**<0.001**	1702	0.003	**0.023**
	Stage	2	NA	NA	NA	2052	0.029	**<0.001**	1700	0.199	**<0.001**
	Habitat	1	4	–	0.11	4	0.018	**<0.001**	4	0.054	**<0.001**
	Locality	4	41	0.086	**<0.001**	1024	0.018	**<0.001**	848	0.017	**<0.001**
	Stage * year	2	NA	NA	NA	2044	–	0.42	1693	0.007	**<0.001**
	Habitat * year	1	8	0.108	**<0.001**	8	0.003	*0.027*	8	0.003	**0.008**
	Locality * year	4	78	0.144	**<0.001**	2036	0.028	**<0.001**	1686	0.008	**0.003**
	Habitat * stage	2	NA	NA	NA	2040	0.006	**0.022**	1690	0.005	**0.004**
	Locality * stage	8	NA	NA	NA	2025	–	0.08	1678	0.008	*0.032*

Significant values (<0.025) are in bold, marginally significant values (<0.05) are in italics. The conventional significance level was reduced by half, due to using a portion of the data in two independent tests (see methods for details). NA indicates values which are not available; the given factor was not tested in the specific case. The R^2^ values represent the proportion of the deviance explained when compared to the total deviance in the data.

Additionally, the species differed in their flowering probabilities, and there were also strong significant differences in flowering probability detected between the two habitat types, with a higher probability of flowering being observed in *A. liliago* and in the open habitats. The probability of remaining in the flowering stage was 72.4% in *A. liliago*, 65.4% in *A. ramosum* from the open habitat and 17.8% in *A. ramosum* from the forest. There were also differences in flowering probabilities between years, and a significant interaction between the species and years was detected (Table 1). The probability of flowering increased by approximately 10% in *A. ramosum* populations in the second year, whereas it decreased in the populations of *A. liliago*. The flowering of the forest populations did not differ between years.

The probability of survival did not differ between the two species in the open habitat. It was, however, significantly lower in the open habitats than in the forest habitats for *A. ramosum* (Table 1). The survival probability for *A. liliago* was 85.8%, while that for *A. ramosum* from the open habitat was 85.5%, and for *A. ramosum* from the forest, it was 73.9%. There was marginally significant effect of the year on survival in the forest populations, with better plant survival being recorded in the second year (Table 1).

### Population Growth Rates

**Figure 2:**
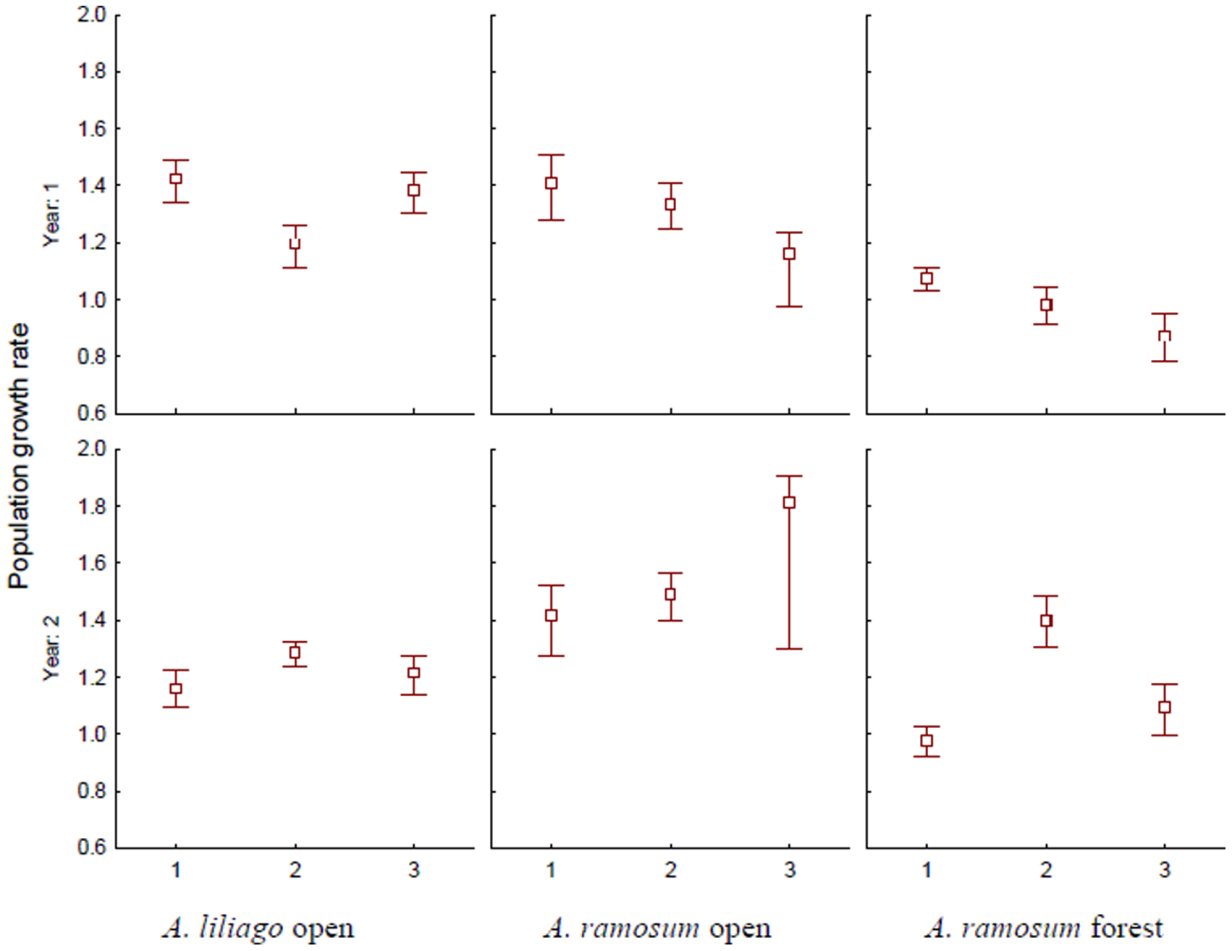
Population growth rate of *A. liliago* and *A. ramosum* in the open and forest habitats, shown separately for each population and transition interval (year 1∶2006–2007, and year 2, 2007–2008), with 95% confidence intervals.

The transition probabilities varied largely both between populations as well as between years (Table S1). Consequently, there was also quite large variation between the single populations and years in terms of the population growth rate (λ) (Figure 2). The values of λ varied between 0.87 and 1.81. One population showed a λ significantly lower than 1; i.e., it was declining (one forest population). Most of the values for the other populations were significantly above 1, i.e., growing.

**Figure 3:**
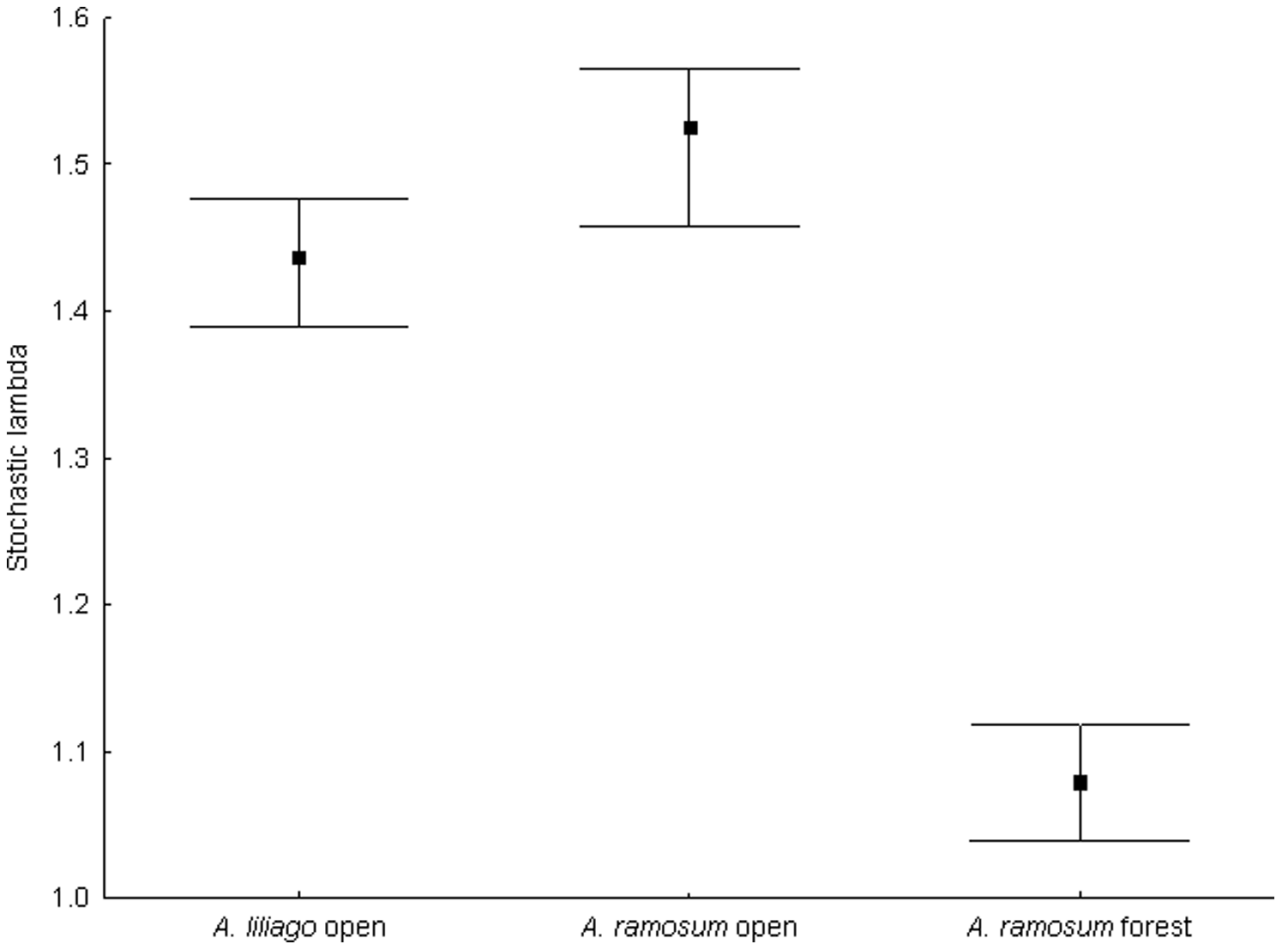
Stochastic population growth rate of *A. liliago* and *A. ramosum* in the open and forest habitats combining data from all populations and years, with 95% confidence intervals.

The stochastic population growth rates in the three population types (the matrices within each type were combined using stochastic simulations) did not differ greatly between *A. liliago* and *A. ramosum* within the open habitats (Figure 3). There were, however, large differences in the stochastic λ between populations of *A. ramosum* from the open habitats and the forest habitats (Figure 3).

### Elasticity

The most important transition for all of the studied population types was transition *a*_33_ (survival of small vegetative plants and clonal growth). The elasticity of this transition was highest in the populations of *A. ramosum* in the forest habitats. Transitions *a*_34_, *a*_43_, *a*_44_ and *a*_45_ (survival and growth) also exhibited quite high elasticity, especially in the forest habitats. Additionally, *A. liliago* displayed high elasticity also in transitions *a*_54_, *a*_55_ and *a*_25_, i.e., the transitions of flowering and reproduction. Similarly, *A. ramosum* from open habitats presented high elasticity for transitions *a*_55_, *a*_15_ and *a*_25_ (i.e., flowering and reproduction). Transitions *a*_21_ and *a*_32_ (i.e., germination and growth) were also very important for *A. ramosum* in the open habitats (Figure S6).

**Figure 4:**
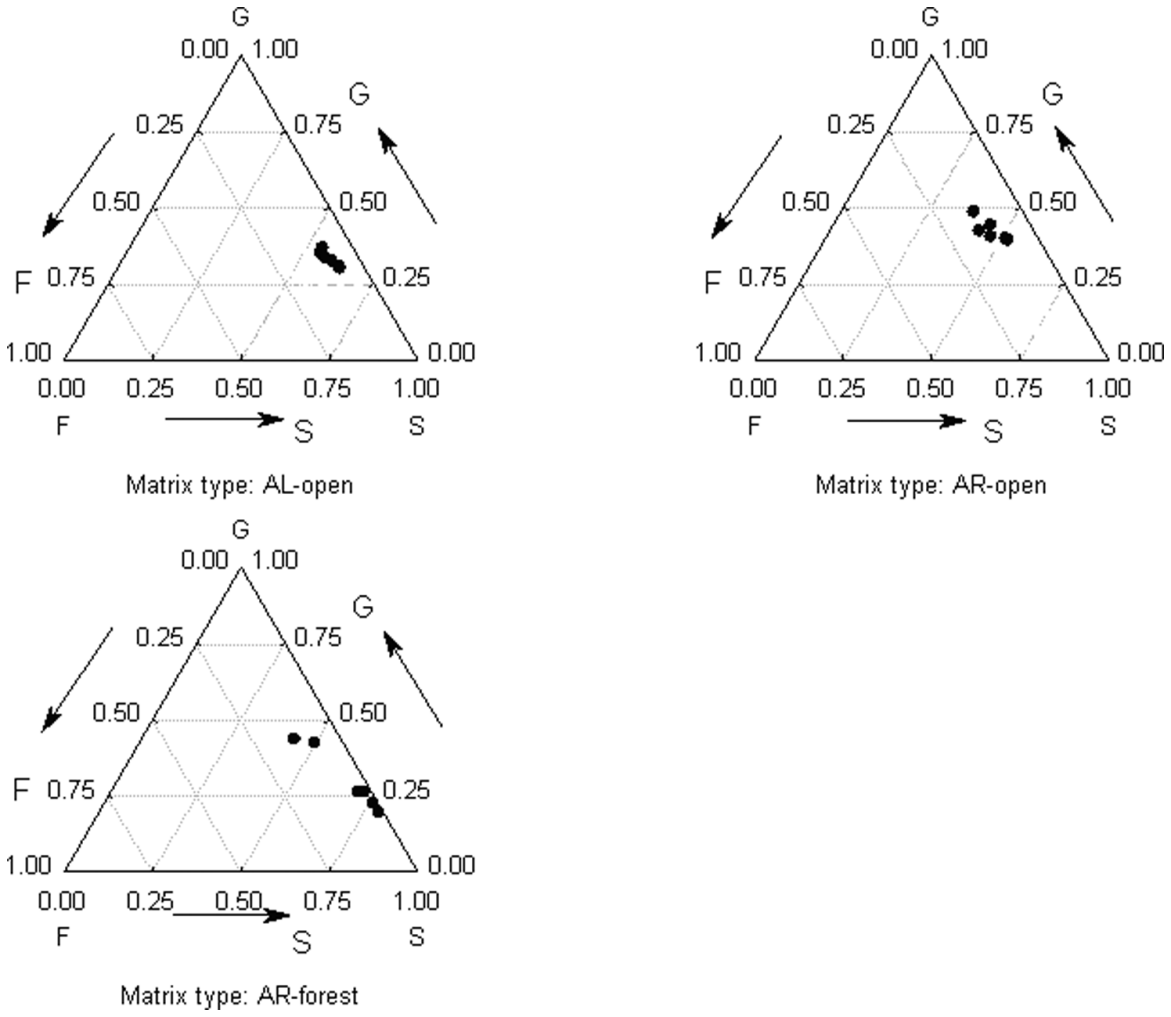
Summed elasticities of fecundity (F), survival, i.e. stasis and retrogression (S) and growth (G) for the three matrix types.

A similar pattern could be seen when the elasticities were summed by survival, growth and fecundity (Figure 4). Among the three examined population types, *A. ramosum* from the open habitats showed the highest elasticity for growth and fecundity, while *A. ramosum* from the forest habitats presented the highest values for survival, and *A. liliago* displayed values between these two populations. Overall, the elasticity of survival showed the highest values in *A. ramosum* from the forest habitats and *A. liliago,* while growth displayed the highest elasticity in *A. ramosum* from the open habitats. In all cases, fecundity was associated with the lowest absolute values of elasticity (Figure 4).

### Life-table Response Experiments

The results of life-table response experiments comparing the population dynamics of *A. liliago* and *A. ramosum* from the open habitats showed that the transitions that significantly contributed to the variation in the population growth rates were germination from the seed bank (transition *a*_21_) and transitions from small and large vegetative plants to flowering plants (*a*_53_, *a*_54_), which all showed higher values in *A. ramosum*. There were also significant contributions of the survival of large vegetative plants (*a*_44_) and survival of seeds in the seed bank (*a*_11_), which were both higher in *A. liliago* (Table 2). The overall contribution of the species to the observed variation in the population growth rates was significant (p = 0.013). The population growth rate was higher overall in *A. ramosum*. Many life-cycle transitions also differed between years and localities. Additionally, there were many significant interactions between species and years (Table 2), and the overall differences between years, localities and the interactions between years and localities were significant (Table 2).

**Table 2:** Results of LTRE analyses comparing overall population dynamics and the contribution of single matrix elements to overall population dynamics for the species *A. ramosum* (AR) and *A. liliago* (AL) in different localities and years.

From stage	To stage	AL	AR	Year 1	Year 2	AL-year1	AL-year2	AR-year1	AR-year2	Locality 1	Locality 2	Locality 3
Seed	Seed	**0.007**	**−0.007**	0.002	−0.002	0.001	0.001	0.007	−0.009	0.003	**−0.021**	**0.018**
Flowering	Seed	0.003	−0.003	0.008	−0.008	−0.011	−0.008	−0.001	**0.020**	**0.030**	**−0.038**	0.008
Seed	Seedling	**−0.030**	**0.030**	**−0.014**	**0.014**	−0.006	−0.006	**−0.012**	**0.024**	**0.017**	**0.072**	**−0.089**
Flowering	Seedling	**−0.017**	**0.017**	−0.005	0.005	−0.005	**−0.019**	**−0.031**	**0.055**	**−0.016**	**−0.018**	**0.034**
Seedling	Small veg.	−0.003	0.003	−0.007	0.007	−0.004	−0.004	**0.011**	−0.004	**−0.011**	0.005	0.006
Small veg.	Small veg.	0.007	−0.007	**0.017**	**−0.017**	**0.034**	**−0.036**	0.005	−0.002	−0.003	−0.002	0.005
Large veg.	Small veg.	0.001	−0.001	0.003	−0.003	0.006	−0.007	−0.001	0.001	−0.002	0.004	−0.002
Flowering	Small veg.	0.000	0.000	0.001	−0.001	0.005	−0.007	0.001	0.002	−0.001	0.001	0.000
Small veg.	Large veg.	0.006	−0.006	−0.005	0.005	0.007	0.010	**−0.029**	0.012	−0.007	−0.006	**0.013**
Large veg.	Large veg.	**0.009**	**−0.009**	0.005	−0.005	0.002	**0.027**	**−0.021**	−0.008	**0.010**	**−0.011**	0.001
Flowering	Large veg.	0.001	−0.001	0.004	−0.004	**−0.015**	**0.013**	**−0.010**	**0.012**	0.007	−0.007	−0.001
Small veg.	Flowering	**−0.020**	**0.020**	**−0.023**	**0.023**	0.004	−0.010	−0.004	**0.010**	**−0.015**	**0.026**	**−0.012**
Large veg.	Flowering	**−0.019**	**0.019**	**−0.013**	**0.013**	−0.009	**−0.047**	**0.033**	**0.023**	**−0.019**	0.004	**0.015**
Flowering	Flowering	−0.001	0.001	−0.008	0.008	**0.032**	**−0.018**	0.006	**−0.020**	**−0.014**	0.003	**0.010**
Overall		**−0.330**	**0.330**	**−0.221**	**0.221**	**0.121**	**−0.332**	**−0.140**	**0.351**	**−0.081**	**0.053**	0.028

The presented values indicate the magnitude of the contribution, with significant values (p≤0.025) shown in bold. For each factor, the contribution to the population growth rate of all levels of the given factor sum to zero. As a result, a positive contribution indicates that the transition increases the overall population growth at the given level of the factor, while a negative contribution indicates that the transition decreases the overall population growth at the given level of the factor. The negative contribution values therefore indicate that this specific transition represents a weak point in the life cycle at the given level of the factor.

The transitions contributing the most to the differences between the populations of *A. ramosum* from the open habitats and the forest habitats were quite different from those responsible for the differences between species. The transitions that contributed most to the variation in population growth rates were the transitions of growth and flowering (*a*_53_, *a*_54_, *a*_55_), which showed significantly higher values in the open habitat (Table 3). Additionally, reproductive transitions *a*_15_ and *a*_25_ contributed to the differences in population dynamics and presented significantly higher values in *A. ramosum* from the open habitats. On the other hand, the survival of large vegetative plants (*a*_44_, *a*_45_) contributed significantly more to the population growth rate in *A. ramosum* from the forest habitats. The overall contribution of the habitat type to the observed differences in the dynamics between the *A. ramosum* populations was highly significant (*p*<0.001), with a positive contribution of the open habitat being detected (Table 3). Many of the life-cycle transitions contributed significantly to the variation between years as well as to the interaction between habitats and years (Table 3). Additionally, the overall contribution of the years and the interaction of habitats and years were significant in all cases. Specifically, the populations performed best overall in the open habitats in the second year and worst in the forest habitats in the first year (Table 3).

**Table 3:** Results of LTRE analyses comparing overall population dynamics and the contribution of single matrix elements to the overall population dynamics of *A. ramosum* in different habitats (open and forest) and years.

		Open	Forest	Year 1	Year 2	Open-year 1	Open-year 2	Forest-year 1	Forest-year 2
Seed	Seed	−0.005	0.005	0.002	−0.002	0.006	−0.009	0.001	0.001
Flowering	Seed	**0.013**	**−0.013**	**0.026**	**−0.026**	**0.012**	0.006	−0.006	**−0.012**
Seed	Seedling	**0.021**	**−0.021**	0.009	−0.009	−0.008	**0.016**	−0.004	−0.004
Flowering	Seedling	**0.008**	**−0.008**	**−0.015**	**0.015**	**−0.023**	**0.072**	**−0.037**	**−0.012**
Seedling	Small veg.	**0.015**	**−0.015**	0.007	−0.007	0.003	**0.030**	**−0.017**	**−0.017**
Small veg.	Small veg.	0.008	−0.008	**−0.010**	**0.010**	0.002	**0.038**	**−0.024**	**−0.016**
Large veg.	Small veg.	0.001	−0.001	−0.001	0.001	−0.001	0.006	−0.005	0.000
Flowering	Small veg.	−0.001	0.001	−0.001	0.001	**0.010**	−0.004	0.001	−0.007
Small veg.	Large veg.	−0.003	0.003	−0.002	0.002	**−0.023**	**0.019**	−0.004	0.008
Large veg.	Large veg.	**−0.004**	**0.004**	−0.008	0.008	**−0.022**	0.004	0.000	**0.018**
Flowering	Large veg.	**−0.009**	**0.009**	−0.002	0.002	**−0.015**	**−0.014**	0.010	**0.020**
Small veg.	Flowering	**0.011**	**−0.011**	0.004	−0.004	**0.011**	**0.013**	−0.001	**−0.023**
Large veg.	Flowering	**0.022**	**−0.022**	**0.014**	**−0.014**	**0.024**	**0.027**	**−0.014**	**−0.037**
Flowering	Flowering	**0.031**	**−0.031**	0.001	−0.001	−0.004	**0.079**	**−0.046**	**−0.029**
Overall		**0.647**	**−0.647**	**0.140**	**−0.140**	**−0.085**	**0.847**	**−0.435**	**−0.327**

The presented values indicate the magnitude of the contribution, with significant values (p≤0.025) shown in bold. For each factor, the contributions to the population growth rate of all levels of the given factor sum to zero. As a result, a positive contribution indicates that the transition increases the overall population growth at the given level of the factor, while a negative contribution indicates that the transition decreases the overall population growth at the given level of the factor. The negative contribution values therefore indicate that that specific transition represents a weak point in the life cycle at the given level of the factor.

**Figure 5:**
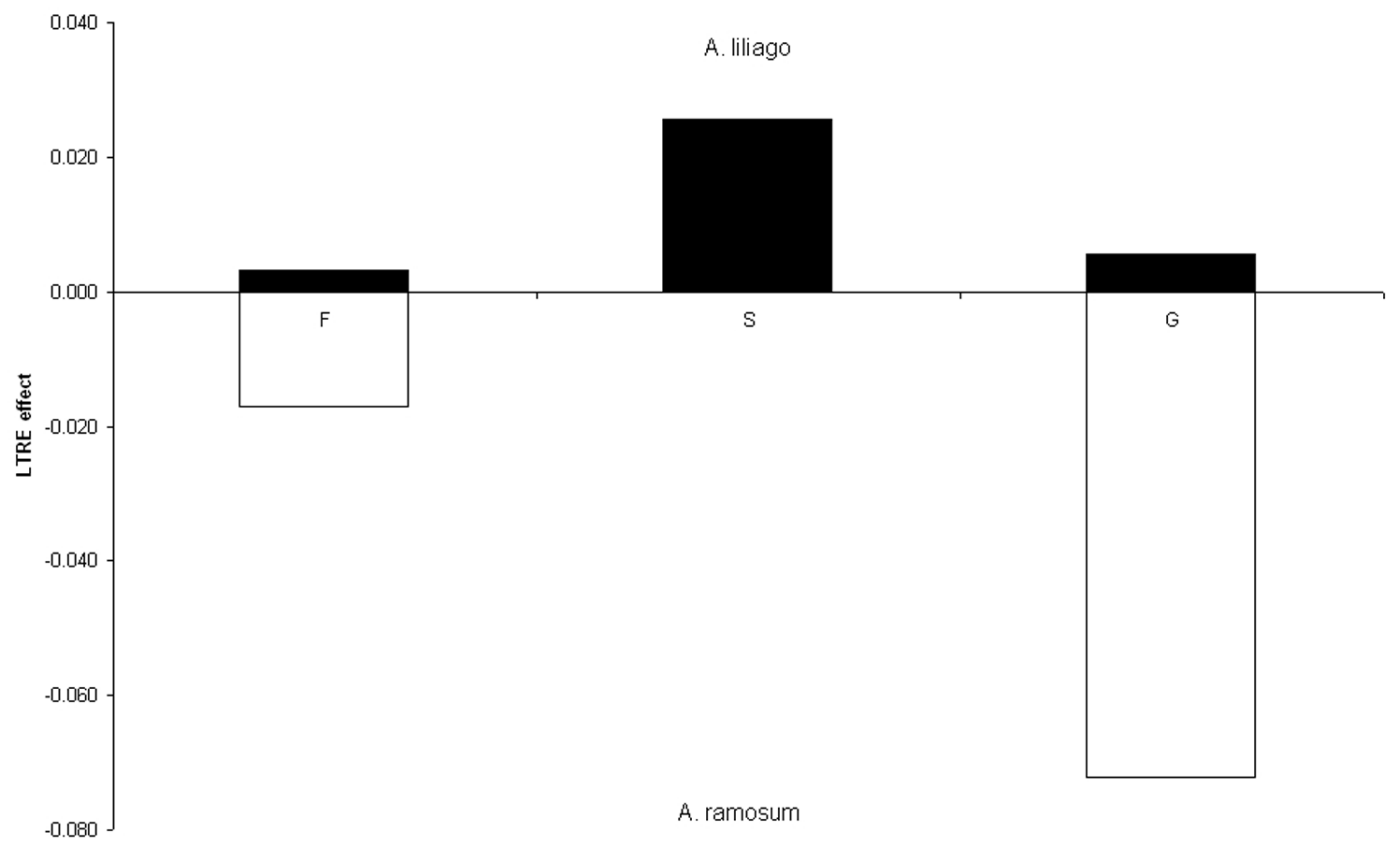
Positive and negative effects grouped by life history components (fecundity (F), survival, i.e. stasis and retrogression (S) and growth (G)) for the three matrix types. Comparison of *A. liliago* and *A. ramosum* from the open habitat.

**Figure 6:**
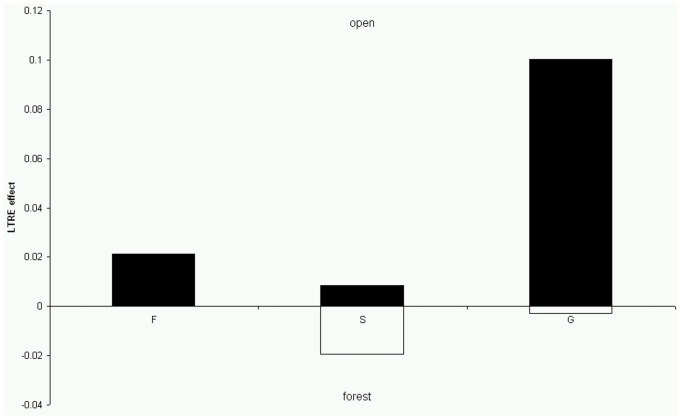
Positive and negative effects grouped by life history components (fecundity (F), survival, i.e. stasis and retrogression (S) and growth (G)) for the three matrix types. Comparison of *A. ramosum* from the open and forest habitats.

Summed LTRE values from comparison between species and between habitat types into those representing survival, growth and reproduction confirmed the previous results. Specifically, the growth of *A. liliago* in contrast to *A. ramosum* from open habitat is mainly mediated by survival transitions. In contrast, growth of *A. ramosum* is mainly mediated by growth related transitions (Figure 5). When comparing *A. ramosum* from the open and forest habitat, growth of populations from open habitat is mainly mediated by growth related transitions while forest populations rely mainly on survival (Figure 6).

### Vital Rate Elasticity

**Figure 7:**
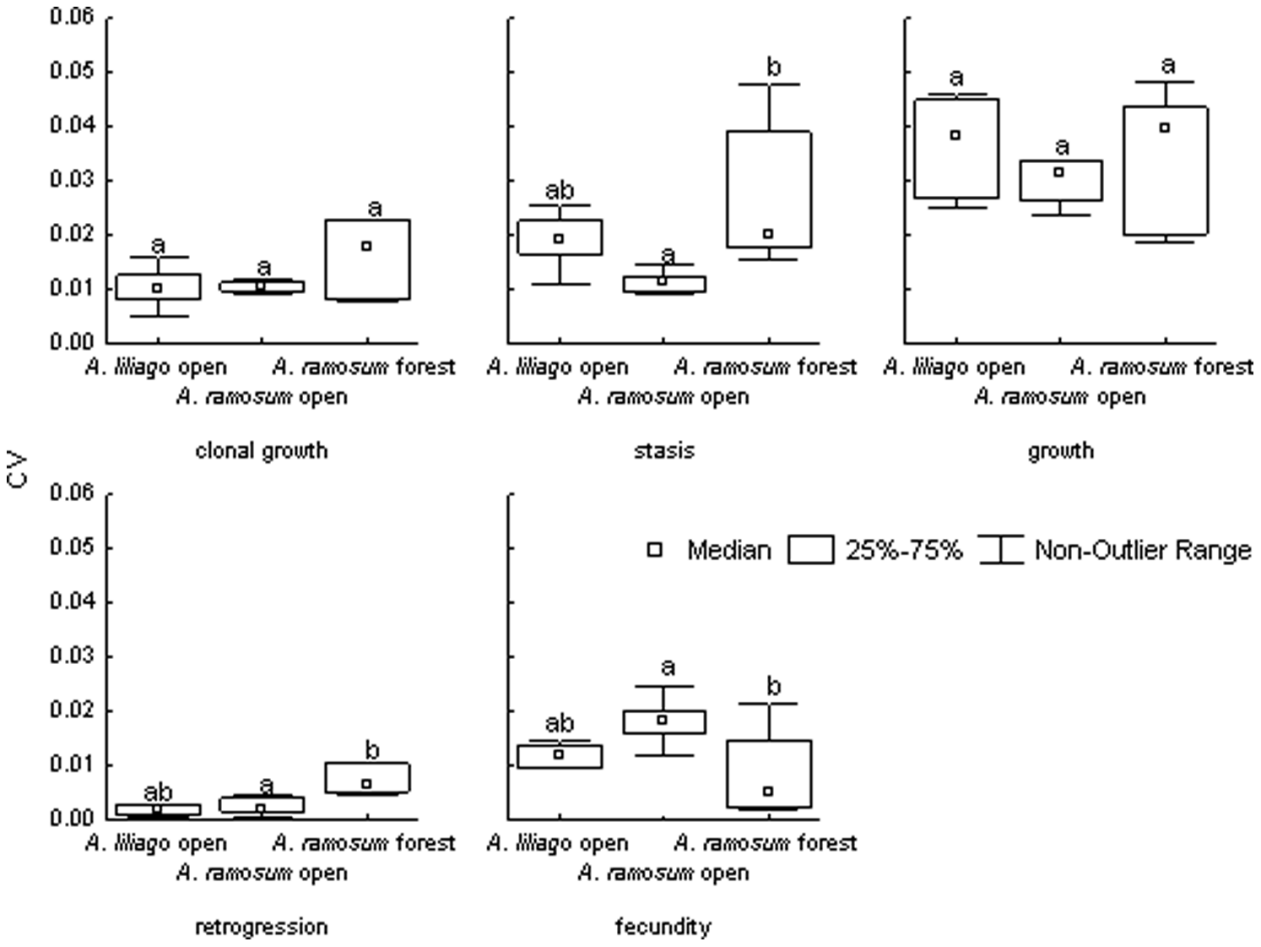
Distribution of coefficients of variation (CV) for lambda from a variance-standardized perturbation analyses for each vital rate. Different letters indicate significant differences (P<0.05).

The vital rates elasticities provide more detailed picture of the importance of single vital rates in the system. Specifically, compared to the elasticity and LTRE, they separate clonal growth that is otherwise part of the stasis transitions in the other analyses. The results showed no differences in the importance of clonal growth and growth between the different matrix types. In contrast, stasis and retrogression are the most important in the forest populations of *A. ramosum* and fecundity is the most important for *A. ramosum* from the open habitat. The *A. liliago* from the open habitats is in between *A. ramosum* from open and forest habitats in terms of stasis, retrogression as well as fecundity (Figure 7).

## Discussion

The results of this study showed that both the single life history traits and mean population dynamics of the studied species, *A. liliago* and *A. ramosum*, from the same (open) habitat type were more similar than between the populations of *A. ramosum* from the two contrasting types of habitat (open and forest). Thus, when transferring knowledge regarding population dynamics between populations, we need to take into account the habitat conditions, as these factors appear to be more important than the species involved (ploidy level).

### Effect of Habitat

The differences in the single life history traits as well as the overall population dynamics between the populations of *A. ramosum* from the open habitat and from the forest were much greater than the differences between the two examined species within a single habitat. This result was detected using analysis of population growth rate, elasticity analysis, life table response experiments and as well as vital rate elasticity. This suggests that this is a robust result independent of a specific analytical technique. Similar large differences in population dynamics between different habitat types within a single species have been found in a range of previous studies (e.g., [34], [35], [36], [37], [67], [21], [68], [42]).

In this study, the population growth rate (λ) of *A. ramosum* from the open habitat was quite high, and these populations are therefore expected to be growing. In contrast, the forest populations of *A. ramosum* showed population growth rates (λ) close to 1, indicating that these populations are rather stable. The higher population growth rate found for *A. ramosum* in the open habitat was linked to the higher individual growth and flowering of plants from the open habitats compared to the forest. These results are in agreement with field observations showing that the populations of *A. ramosum* in the open habitat are large and perform well. In contrast, the forest populations appear to be stable but are not expanding and are even declining in some places.

The results of this study also showed strong differences in population dynamics between years. The extent of temporal variation was, in fact, comparable to the extent of spatial variation, suggesting that information on spatial variation can be used to infer patterns of temporal variation, as reported, e.g., by [37].

The large differences in species dynamics between the two habitat types can be explained by the differences in the conditions in these habitat types. The open habitats are characterized by sparse vegetation, associated with numerous opportunities for the establishment of new individuals. Due to the high availability of light and sufficient availability of nutrients and moisture, the plants at these sites tend to grow rapidly and, thus, reproduce relatively early in their life cycle. In contrast, generative reproduction in the forest sites is limited by the absence of safe sites for germination due to the presence of dense understory vegetation and/or a deep needle layer. The plants in the forests are also likely to suffer from a lack of light and limited availability of moisture, which is taken up by the trees. A clear advantage of the forest environment is its stability, including a limited occurrence of external disturbances and low fluctuations in climatic conditions. Conditions such as these, which are unfavorable overall, but stable over time, favor individuals that grow slowly and invest mainly in survival and/or clonal growth. In contrast, the conditions in the open habitats are expected to be much more variable. Even though it was not captured within our study period, the open sites, which are usually situated on steep slopes, experience frequent disturbances due to the movement of the substrate as well as the activity of wild ungulates. In addition, the open sites are much more likely to be subjected to various types of weather extremes, such as extreme droughts or frosts, which may cause substantial plant mortality and, thus, open further opportunities for the establishment of new individuals.

These differences in species performance among the different habitat types are also clearly visible from the elasticity analyses, LTRE analyses as well as vital rate elasticities. Generative reproduction and germination from the seed bank were more important for population growth in *A. ramosum* in the open habitats, while survival transitions, including clonal growth, were more important for *A. ramosum* in the forests. As indicated by the vital rates elasticity, the clonal growth per se, however, did not significantly differ between the habitat types. Overall, the obtained elasticity values were comparable to those reported for similar species in similar habitat types, with a greater importance of survival being observed in forests than in open habitats (e.g., [69], [60], [70]).

In this study, we followed populations in two different environments and followed only plants native to each of the environment in each case. We do not have any information on the genetic relationships between the plants in the two different environments nor on the importance of local adaptation in the system. As plants in different environments may be indeed locally adapted and genetically differentiated (e.g. [71], [72]) the differences between the two environments need to be interpreted as the differences between the populations in these environments and it is not possible to separate the effects of environment per se from the effects from the population type per se. While it would be ideal to observe population dynamics after transplanting individuals between the environments [31], this is only feasible in short-lived species. Thus, most studies exploring plant population dynamics in different environments indeed work with local populations only (e.g., [34], [35], [36], [37], [15], [39], [40], [41], [42], [43]).

### Effects of Species

In contrast to the relatively large number of studies comparing populations of the same species in different habitat types, relatively few studies have compared the population dynamics of closely related species or cytotypes of a single species. In one of these studies, Münzbergová [15] showed that the differences in population dynamics between two ploidy levels were quite small. In contrast, Bucharová et al. [21] reported large differences between two closely related fern species, with a higher extinction probability and smaller number of populations being found for a diploid species compared to a tetraploid species. Similarly, Buggs & Pannell [31] demonstrated higher fitness in diploids compared to tetraploids. Finally, Münzbergová [22] showed strong differences in population dynamics of two closely related *Linum* species growing on the same habitats.

In this study, the population growth rate of the diploid species *A. ramosum* in the open habitat was significantly higher than the population growth rate of the allotetraploid species *A. liliago* in the same habitat, as indicated by the LTRE analysis. This result suggests that the species (ploidy levels) involved have significant consequences for population dynamics. In addition, the elasticity and LTRE analyses demonstrated that generative reproduction and germination from the seed bank were more important for population growth in *A. ramosum* in the open habitats, while flowering was more important for population growth in *A. liliago* in the same habitats. Indication of similar patterns can be seen also from the vital rates elasticity. In this case, however, the differences between *A. liliago* and *A. ramosum* from the open habitat are never significant.

There are several possible explanations for the differences in population dynamics observed between the species. First, there could be differences related to the local habitat conditions experienced by each species. However, the microhabitats occupied by the two species within the open localities were not found to differ in the present study, and the differences in the population dynamics between the two species therefore must be explained by their biological traits.

Overall, the morphology of the two species is very similar, as are their growth strategies [73]. One of the important differences between the studied species is the difference in their phenology. *A. liliago* flowers much earlier than *A. ramosum* and is therefore likely to experience different climatic conditions within the season. Indeed, the between-year variation in the performance of the two species was not correlated. According to the LTRE analyses, *A. liliago* performed significantly better in the first (wetter) transition period, while *A. ramosum* performs significantly better in the second (drier) transition period.

Another important difference between these species is linked to their ploidy level, as *A. ramosum* is diploid, and *A. liliago* is allotetraploid [51]. Large differences between diploids and tetraploids have been repeatedly suggested in many studies comparing single life history traits (e.g., [45], [46], [47], [48], [49]) and the overall species dynamics ([31], [21]) of diploids and polyploids. The general pattern observed and expected is that polyploid species perform better than their diploid ancestors because of possessing a greater number of different alleles and, thus, being able to adapt to different environments better (e.g., [44], [45],[46], [47], [48], [49]). However, some studies have not found significant differences between cytotypes (e.g., [74], [50], [15], [56]). In this study, we observed a higher population growth rate in the diploid species than in the tetraploid species. This finding is in agreement with the wider range of habitats occupied by the diploid species *A. ramosum* in our study region. This observation is also in agreement with conclusions of Buggs & and Pannell [31] indicating that diploids of *Mercurialis annua* perform better than tetraploids.

When interpreting the differences between the two species, it is important to keep in mind that the two species differ in multiple aspects. In addition to being different species, the two study units also represent two different cytotypes. In addition, the allotetraploid *A. liliago* is a hybrid species. This study thus does not allow us to tell, if the differences between the two units are differences between species, cytotypes or are due to the hybrid origin of one of them.

### Overall Comparison

Comparison of all three population types suggested that *A. ramosum* from the forest was more similar in some aspects to *A. liliago* than to *A. ramosum* from the open habitats. When arranged into the G-L-F (growth-survival-fecundity) space according to their elasticity values [60], *A. ramosum* from the open habitats fell among other perennial herbs from open habitats, while *A. ramosum* from the forest habitats fell among other perennial herbs from forest habitats. *A. liliago* fell in between these two groups. Interestingly, *A. liliago* performed better in the wetter transition interval, while *A. ramosum* from the open habitat performed better in the drier transition interval. *A. ramosum* from the forest did not show any consistent between year variation. Given that the forests are indeed wetter than the open habitats, this seem that *A. ramosum* from the open habitats is adapted to very different conditions than *A. ramosum* from the forests. Based on all the above it therefore appears that the populations of the diploid *A. ramosum* from the open habitats and the forest habitats represent two specialized ecotypes, while the allotetraploid *A. liliago* is a rather generalist type of species, showing a wider range of growth possibilities.

The differences in elasticity values detected between the studied species and habitats reflect the observed differences in population growth rates. Specifically, the populations from the open habitats grow more vigorously and show a greater reliance on generative reproduction. On the other hand, the forest populations of *A. ramosum* exhibit a much lower population growth rate and higher survival elasticity. Similar correlations between elasticity and population growth rates have previously been shown in both within- and between-species comparisons (e.g., [34], [22]). These authors also recorded higher population growth rates in species relying more generative reproduction and lower population growth rates in species relying on survival. Similar differences between the 3 types of populations were also confirmed by LTRE analyses and more detailed analysis of vital rate elasticities. This confirms that this conclusion is rather robust and is not dependent on specific technique used to analyze the data.

In this study, we compared both of the cytotypes in their native localities and took the advantage from the fact that the two species co-occur at the open habitat type. Only *A. ramosum* was, however, studied at the forest habitats. We are thus not able to compare performance of the two species at both habitat types. Two previous studies, Flegrová and Krahulec [75] and Buggs & Pannell [31], performed reciprocal transplant experiments between habitats occupied by diploids and tetraploids and thus could separately test the effects of ploidy level and of the habitat type. The reason for the current design of our study is that we wanted to study full life cycle of these long-lived clonally growing species and doing so in a reciprocal transplant experiment would be extremely difficult. The two previous studies mentioned above in fact studied only growth and flowering of transplanted ramets in case of Flegrová and Krahulec [75] and fitness of an annual plant in case Buggs & Pannell [31], which is much less complex.

### Between Year Variation

The studied years differed mainly in precipitations, with 2006 and 2007 being close to the average, and 2008 being below average. Similar low values are, however, not exceptional and values lower or equal to that of 2008 occur in 27% of years. This has two important implications. First, the frequency of years with climate similar to one and the other transition intervals is comparable, and calculating stochastic population growth rate assuming the same probability of each year is sensible (see e.g. [64], [65] for models assuming different frequency of different years). Second, the climatic data in fact suggest, that the range of climatic conditions covered within the study is relatively small compared to the climatic variation over the last 50 years. The range of values of mean annual precipitation in fact covers only 20% of the range in the last 50 years. This thus suggests that we did not capture the extreme years. It is thus possible that the conclusions of this study could be altered if using more transition periods (e.g. [76]–[78]). The conclusions of this study should thus be interpreted with caution. While the conclusion that the habitats are more different than species can be considered as valid, the real values of population growth rate should be mainly taken as possible values out of wider range of real values in the field. For the variation between habitats, it can be in fact assumed that more climatically extreme years, especially years much drier, would lead to even larger differences between habitats.

Different life stages respond differently to between year variation [79] and especially seed germination and seedling establishment is known to strongly vary between years (e.g. [42], [80], [81]. In our data, we have data on seed germination from two transition intervals and data on seedling survival even only from one. This is clearly very limited information on this crucial part of the life cycle of the species and is likely the weakest point of this study. We, however, assume that the general message holds even with this limitation as discussed above.

## Conclusions

The results of this study showed that single life history traits as well as the mean population dynamics of the studied allotetraploid species *A. liliago* and diploid species *A. ramosum* in the same (open) habitat type were more similar than the population dynamics of *A. ramosum* from two contrasting habitat types. The results therefore suggest that when transferring knowledge regarding population dynamics between populations, we need to take habitat conditions into account, as these conditions appear to be more important than the species (ploidy level) involved. However, despite being smaller than the differences between the two habitats, the overall population growth rates did show significant differences between the two species (ploidy levels). In contrast to what has been suggested in previous studies, we observed a higher population growth rate in the diploid than in the tetraploid species. This observation is in agreement with the wider range of habitats occupied by the diploid species.

## Supporting Information

Figure S1 PCA of the vegetation composition in the studied localities and populations. Graph shows position of the samples. The first axis explained 38.7% and the second axis 22.5% of total variation in the dataset. The 3 types of studied populations (AL open, AR open, AR forest) are distinguished by the shapes of symbols. The 6 studied localities are distinguished by color.
(TIF)

Figure S2 PCA of the vegetation composition in the studied localities and populations. Graph shows position of the species. The first axis explained 38.7% and the second axis 22.5% of total variation in the dataset. The 31 species contributing most to the variation are shown.
(TIF)

Figure S3 PCA of the soil conditions in the studied localities and populations. The first axis explained 54.3% and the second axis 27.3% of the total variation in the dataset. The 3 types of studied populations (AL open, AR open, AR forest) are distinguished by the shapes of symbols. The 6 studied localities are distinguished by color.
(TIF)

Figure S4 Mean annual temperature in the studied region (central Bohemia, Czech Republic) between 1961 and 2012. The studied years 2006–2008 are show with large symbols. The dashed line indicates mean values of the whole period. The data were obtained from Czech Hydrometerological Institute, www.chmi.cz.
(TIF)

Figure S5 Annual precipitation in the studied region (central Bohemia, Czech Republic) between 1961 and 2012. The studied years 2006–2008 are show with large symbols. The dashed line indicates mean values of the whole period. The data were obtained from Czech Hydrometerological Institute, www.chmi.cz.
(TIF)

Figure S6 Mean stochastic elasticity of single life-cycle transitions in the three population types of *A. liliago* (LO) and in *A. ramosum* from open (RO) and forest (RF) habitat, with 95% confidence intervals.
(TIF)

Table S1 Transition matrices for each transition interval and population. Locality numbers correspond to Figure 1.
(DOC)
