# Supplementary figures and images for: Comparative Population Dynamics of Two Closely Related Species Differing in Ploidy Level
Source: PLoS One. 2013 Oct 7;8(10):e75563. doi: 10.1371/journal.pone.0075563 (PMC3792132; doi:10.1371/journal.pone.0075563)

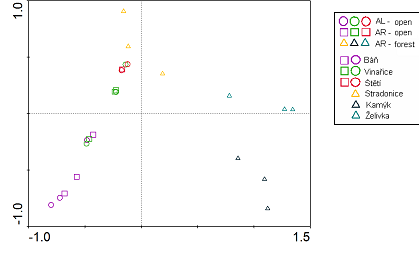

Supplement: Figure S1 — PCA of the vegetation composition in the studied localities and populations. Graph shows position of the samples. The first axis explained 38.7% and the second axis 22.5% of total variation in the dataset. The 3 types of studied populations (AL open, AR open, AR forest) are distinguished by the shapes of symbols. The 6 studied localities are distinguished by color. (TIF) [file pone.0075563.s001.tif]

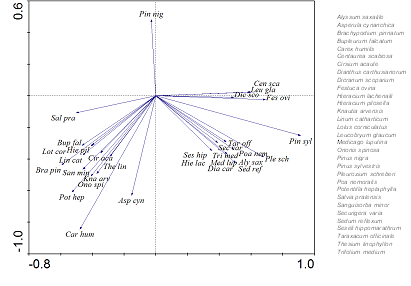

Supplement: Figure S2 — PCA of the vegetation composition in the studied localities and populations. Graph shows position of the species. The first axis explained 38.7% and the second axis 22.5% of total variation in the dataset. The 31 species contributing most to the variation are shown. (TIF) [file pone.0075563.s002.tif]

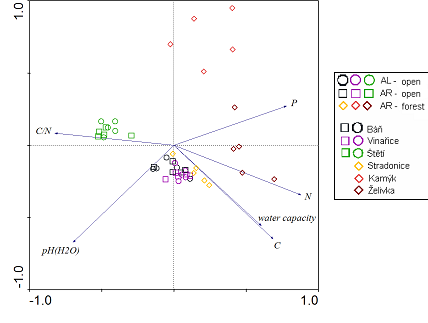

Supplement: Figure S3 — PCA of the soil conditions in the studied localities and populations. The first axis explained 54.3% and the second axis 27.3% of the total variation in the dataset. The 3 types of studied populations (AL open, AR open, AR forest) are distinguished by the shapes of symbols. The 6 studied localities are distinguished by color. (TIF) [file pone.0075563.s003.tif]

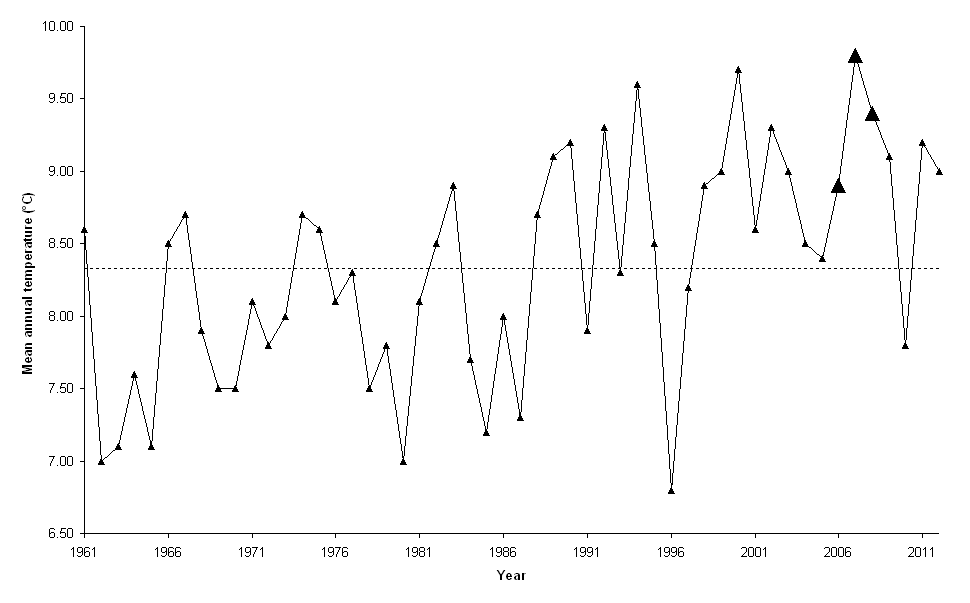

Supplement: Figure S4 — Mean annual temperature in the studied region (central Bohemia, Czech Republic) between 1961 and 2012. The studied years 2006–2008 are show with large symbols. The dashed line indicates mean values of the whole period. The data were obtained from Czech Hydrometerological Institute, www.chmi.cz. (TIF) [file pone.0075563.s004.tif]

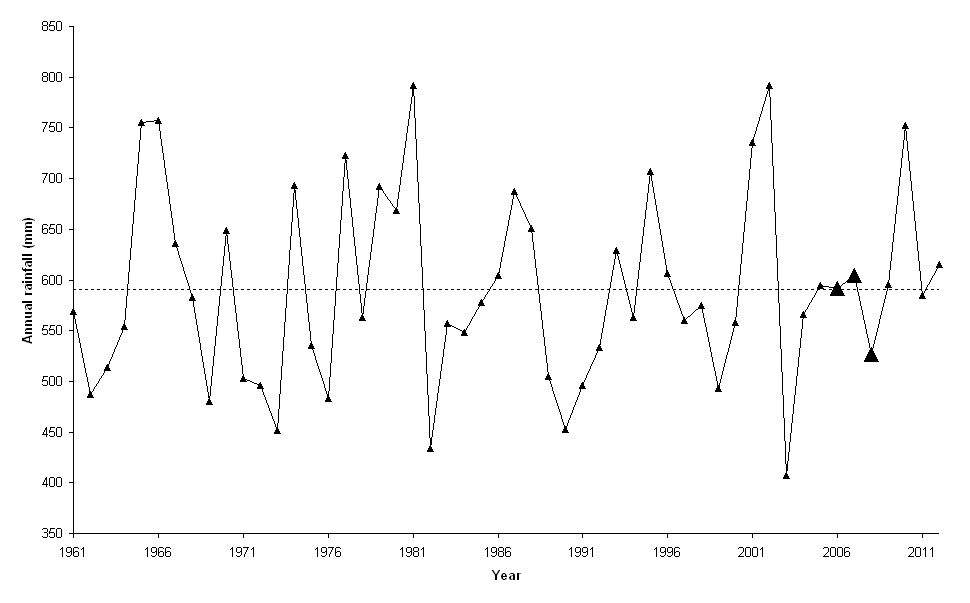

Supplement: Figure S5 — Annual precipitation in the studied region (central Bohemia, Czech Republic) between 1961 and 2012. The studied years 2006–2008 are show with large symbols. The dashed line indicates mean values of the whole period. The data were obtained from Czech Hydrometerological Institute, www.chmi.cz. (TIF) [file pone.0075563.s005.tif]

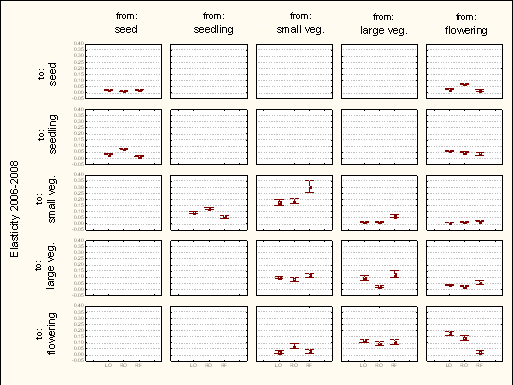

Supplement: Figure S6 — Mean stochastic elasticity of single life-cycle transitions in the three population types of A. liliago (LO) and in A. ramosum from open (RO) and forest (RF) habitat, with 95% confidence intervals. (TIF) [file pone.0075563.s006.tif]
