# Supplementary material for: Comparative Population Dynamics of Two Closely Related Species Differing in Ploidy Level
Source: PLoS One. 2013 Oct 7;8(10):e75563. doi: 10.1371/journal.pone.0075563 (PMC3792132; doi:10.1371/journal.pone.0075563)
Supplement: Table S1 — Transition matrices for each transition interval and population. Locality numbers correspond to Figure 1. (DOC) [file pone.0075563.s007.doc]

Table S1. Transition matrices for each transition interval and population. Locality numbers correspond to Figure 1.

|  |  |  |  | 2006-2007 | | | | | 2007-2008 | | | | |
| --- | --- | --- | --- | --- | --- | --- | --- | --- | --- | --- | --- | --- | --- |
| Loc. no. | Species | Hab. type |  | Seed | Seedl. | Small veg. | Large veg. | Flow. | Seed | Seedl. | Small veg. | Large veg. | Flow. |
| 1 | A. liliago | Open | Seed | 0.63 | 0 | 0 | 0 | 17.222 | 0.63 | 0 | 0 | 0 | 17.889 |
|  |  |  | Seedling | 0.05 | 0 | 0 | 0 | 2.393 | 0.05 | 0 | 0 | 0 | 1.726 |
|  |  |  | Small veg. | 0 | 0.667 | 1.076 | 0.36 | 0.340 | 0 | 0.667 | 0.553 | 0.049 | 0.036 |
|  |  |  | Large veg. | 0 | 0 | 0.179 | 0.551 | 0.245 | 0 | 0 | 0.207 | 0.692 | 0.592 |
|  |  |  | Flowering | 0 | 0 | 0 | 0.381 | 0.733 | 0 | 0 | 0 | 0.274 | 0.400 |
| 2 |  |  | Seed | 0.46 | 0 | 0 | 0 | 1.831 | 0.46 | 0 | 0 | 0 | 1.662 |
|  |  |  | Seedling | 0.08 | 0 | 0 | 0 | 0.516 | 0.08 | 0 | 0 | 0 | 0.685 |
|  |  |  | Small veg. | 0 | 0.967 | 0.667 | 0.296 | 0.200 | 0 | 0.967 | 0.638 | 0.118 | 0.094 |
|  |  |  | Large veg. | 0 | 0 | 0.269 | 0.621 | 0.198 | 0 | 0 | 0.316 | 0.414 | 0.122 |
|  |  |  | Flowering | 0 | 0 | 0.096 | 0.167 | 0.741 | 0 | 0 | 0.07 | 0.548 | 0.873 |
| 3 |  |  | Seed | 0.64 | 0 | 0 | 0 | 5.728 | 0.64 | 0 | 0 | 0 | 5.872 |
|  |  |  | Seedling | 0.05 | 0 | 0 | 0 | 1.504 | 0.05 | 0 | 0 | 0 | 1.360 |
|  |  |  | Small veg. | 0 | 0.867 | 0.904 | 0.297 | 0.245 | 0 | 0.867 | 0.778 | 0.131 | 0.133 |
|  |  |  | Large veg. | 0 | 0 | 0.176 | 0.308 | 0.086 | 0 | 0 | 0.165 | 0.611 | 0.240 |
|  |  |  | Flowering | 0 | 0 | 0.035 | 0.59 | 0.886 | 0 | 0 | 0 | 0.333 | 0.737 |
| 1 | A. ramosum | Open | Seed | 0.02 | 0 | 0 | 0 | 6.663 | 0.02 | 0 | 0 | 0 | 6.474 |
|  |  |  | Seedling | 0.69 | 0 | 0 | 0 | 0.595 | 0.69 | 0 | 0 | 0 | 0.784 |
|  |  |  | Small veg. | 0 | 0.833 | 0.872 | 0.431 | 0.273 | 0 | 0.833 | 0.612 | 0.225 | 0.231 |
|  |  |  | Large veg. | 0 | 0 | 0.148 | 0.279 | 0.325 | 0 | 0 | 0.169 | 0.314 | 0.138 |
|  |  |  | Flowering | 0 | 0 | 0.066 | 0.471 | 0.553 | 0 | 0 | 0.108 | 0.476 | 0.750 |
| 2 |  |  | Seed | 0.08 | 0 | 0 | 0 | 5.096 | 0.08 | 0 | 0 | 0 | 3.688 |
|  |  |  | Seedling | 0.31 | 0 | 0 | 0 | 1.306 | 0.31 | 0 | 0 | 0 | 2.714 |
|  |  |  | Small veg. | 0 | 0.833 | 0.823 | 0.395 | 0.295 | 0 | 0.833 | 0.823 | 0.289 | 0.202 |
|  |  |  | Large veg. | 0 | 0 | 0.237 | 0.421 | 0.306 | 0 | 0 | 0.153 | 0.313 | 0.189 |
|  |  |  | Flowering | 0 | 0 | 0.031 | 0.368 | 0.583 | 0 | 0 | 0.102 | 0.571 | 0.774 |
| 3 |  |  | Seed | 0.65 | 0 | 0 | 0 | 15.961 | 0.65 | 0 | 0 | 0 | 10.917 |
|  |  |  | Seedling | 0.04 | 0 | 0 | 0 | 0.000 | 0.04 | 0 | 0 | 0 | 5.044 |
|  |  |  | Small veg. | 0 | 1 | 0.697 | 0.279 | 0.256 | 0 | 1 | 0.938 | 0.333 | 0.339 |
|  |  |  | Large veg. | 0 | 0 | 0.121 | 0.143 | 0.284 | 0 | 0 | 0.203 | 0.191 | 0.123 |
|  |  |  | Flowering | 0 | 0 | 0.076 | 0.619 | 0.493 | 0 | 0 | 0.116 | 0.8 | 0.882 |
| 4 | A. ramosum | Forest | Seed | 0.39 | 0 | 0 | 0 | 1.601 | 0.39 | 0 | 0 | 0 | 0.952 |
|  |  |  | Seedling | 0.07 | 0 | 0 | 0 | 0.099 | 0.07 | 0 | 0 | 0 | 0.748 |
|  |  |  | Small veg. | 0 | 0.615 | 0.818 | 0.316 | 0.308 | 0 | 0.615 | 0.728 | 0.409 | 0.122 |
|  |  |  | Large veg. | 0 | 0 | 0.173 | 0.534 | 0.727 | 0 | 0 | 0.182 | 0.566 | 0.711 |
|  |  |  | Flowering | 0 | 0 | 0.037 | 0.241 | 0.091 | 0 | 0 | 0 | 0.075 | 0.158 |
| 5 |  |  | Seed | 0.61 | 0 | 0 | 0 | 10.300 | 0.61 | 0 | 0 | 0 | 5.438 |
|  |  |  | Seedling | 0.08 | 0 | 0 | 0 | 0.000 | 0.08 | 0 | 0 | 0 | 4.862 |
|  |  |  | Small veg. | 0 | 0.558 | 0.496 | 0.217 | 0.072 | 0 | 0.558 | 0.634 | 0.228 | 0.158 |
|  |  |  | Large veg. | 0 | 0 | 0.212 | 0.385 | 0.626 | 0 | 0 | 0.354 | 0.475 | 0.286 |
|  |  |  | Flowering | 0 | 0 | 0.03 | 0.333 | 0.045 | 0 | 0 | 0.063 | 0.327 | 0.636 |
| 6 |  |  | Seed | 0.55 | 0 | 0 | 0 | 1.750 | 0.55 | 0 | 0 | 0 | 0.756 |
|  |  |  | Seedling | 0.07 | 0 | 0 | 0 | 0.000 | 0.07 | 0 | 0 | 0 | 0.994 |
|  |  |  | Small veg. | 0 | 0.5 | 0.675 | 0.326 | 0.569 | 0 | 0.5 | 0.828 | 0.365 | 0.448 |
|  |  |  | Large veg. | 0 | 0 | 0.132 | 0.188 | 0.295 | 0 | 0 | 0.176 | 0.44 | 0.541 |
|  |  |  | Flowering | 0 | 0 | 0.044 | 0.317 | 0.000 | 0 | 0 | 0.024 | 0.28 | 0.135 |
